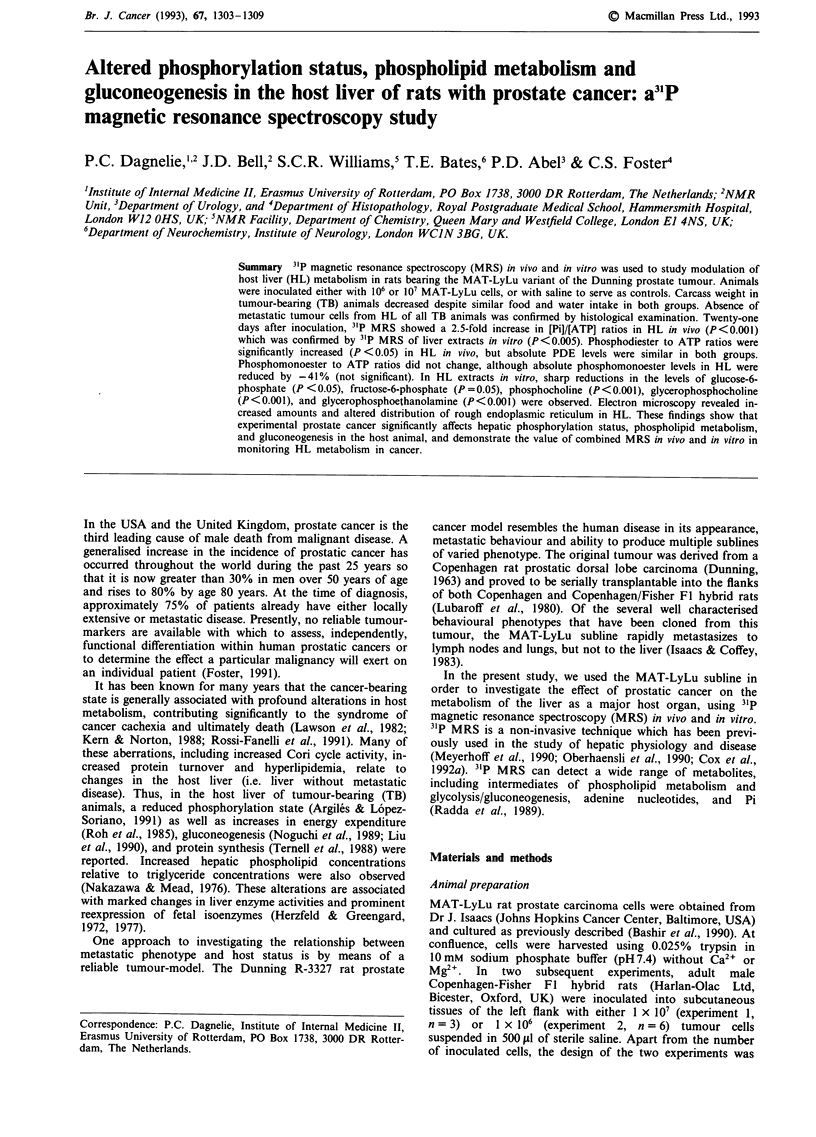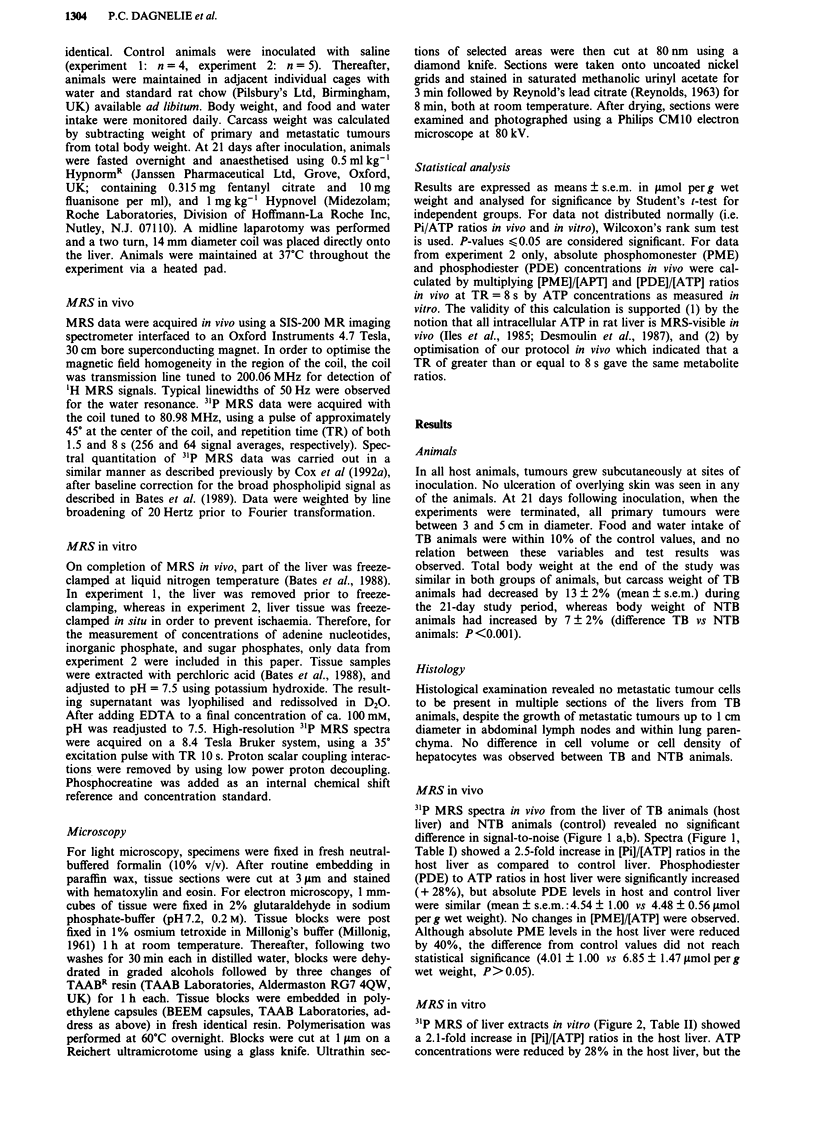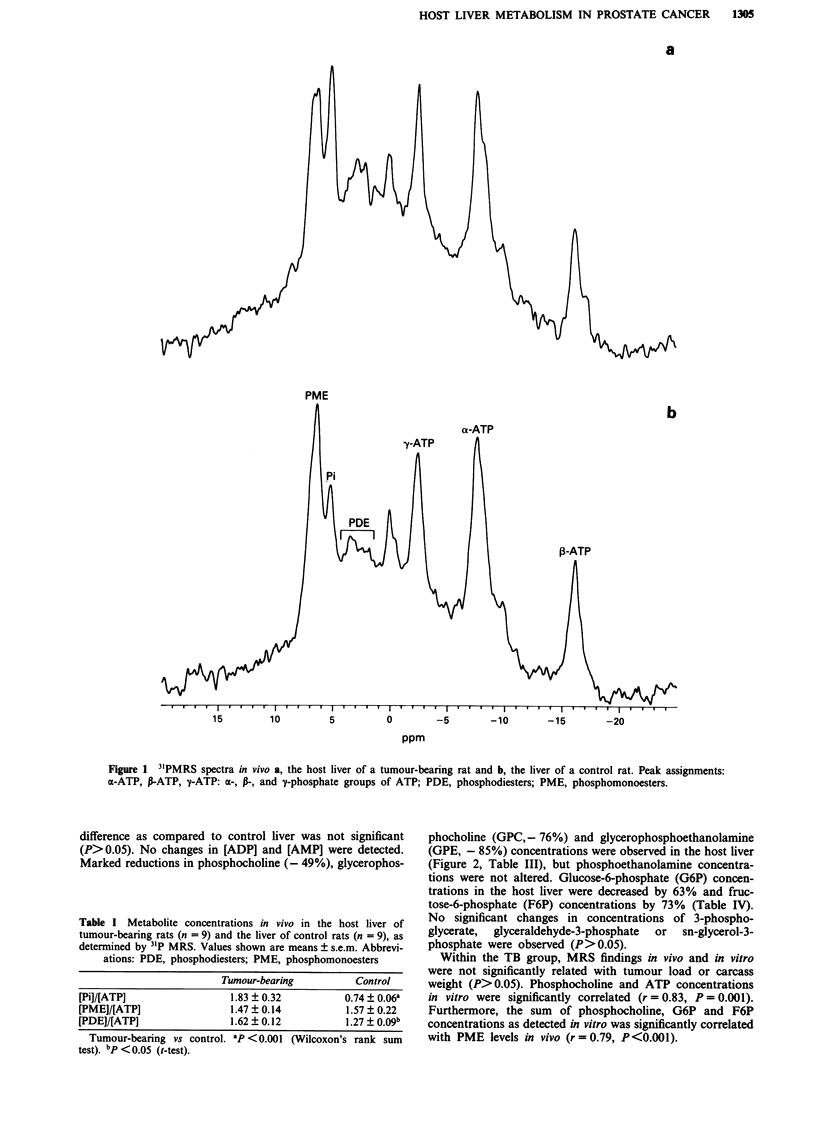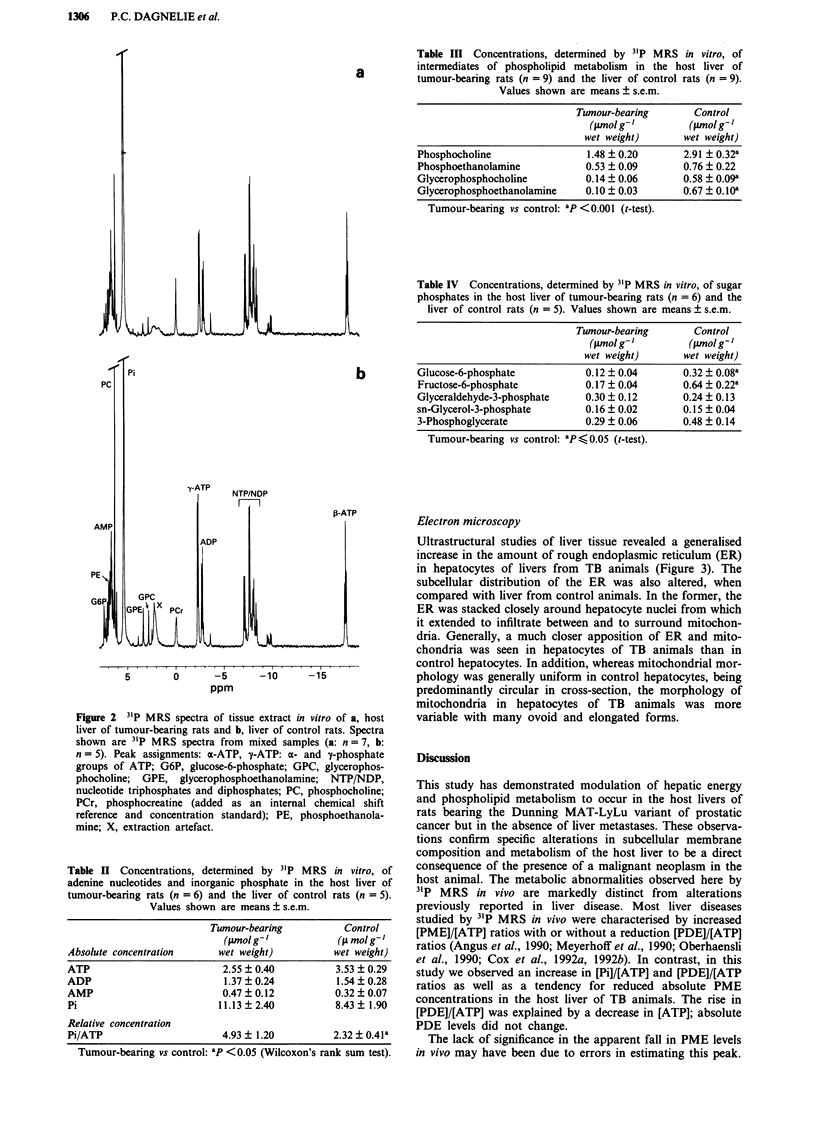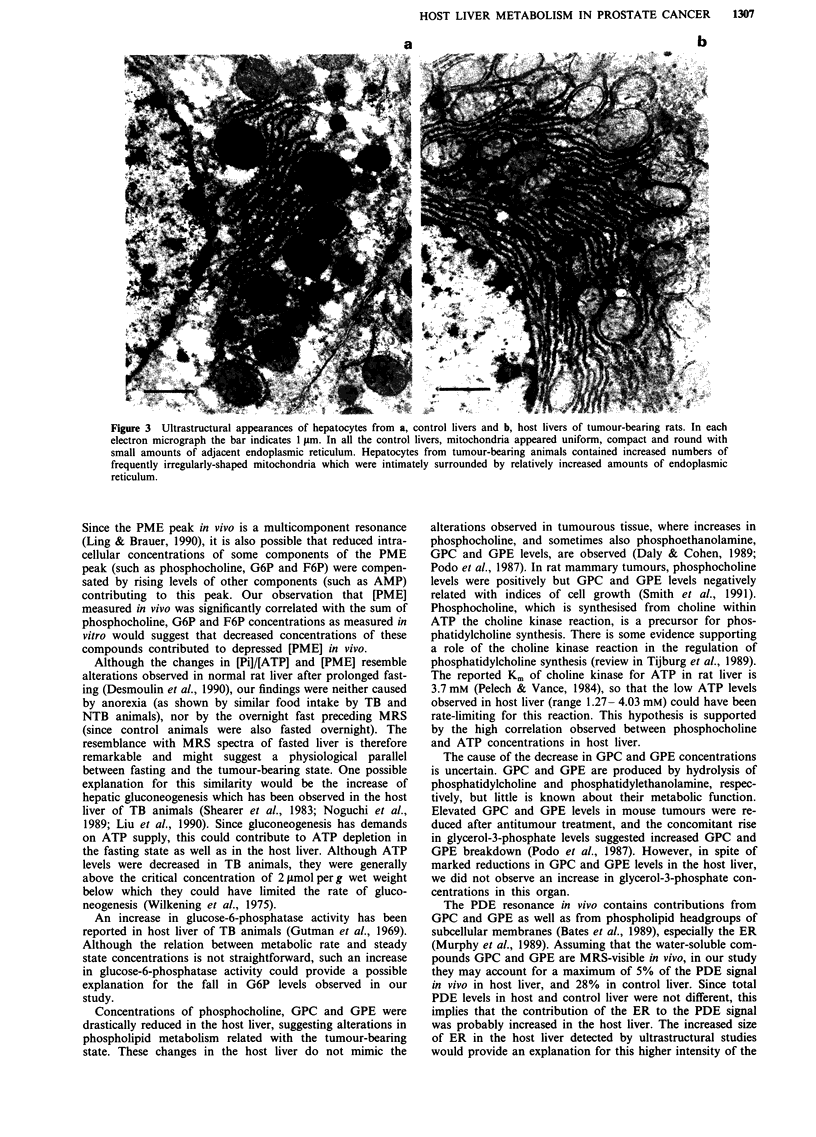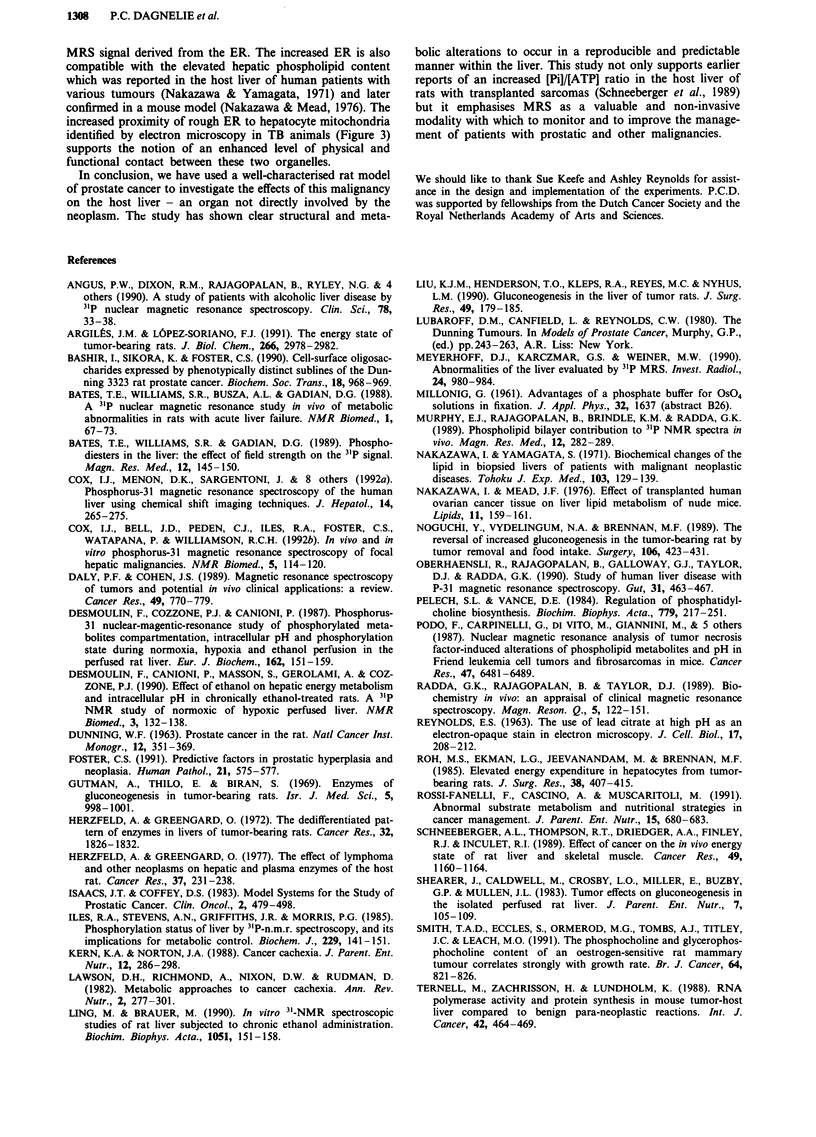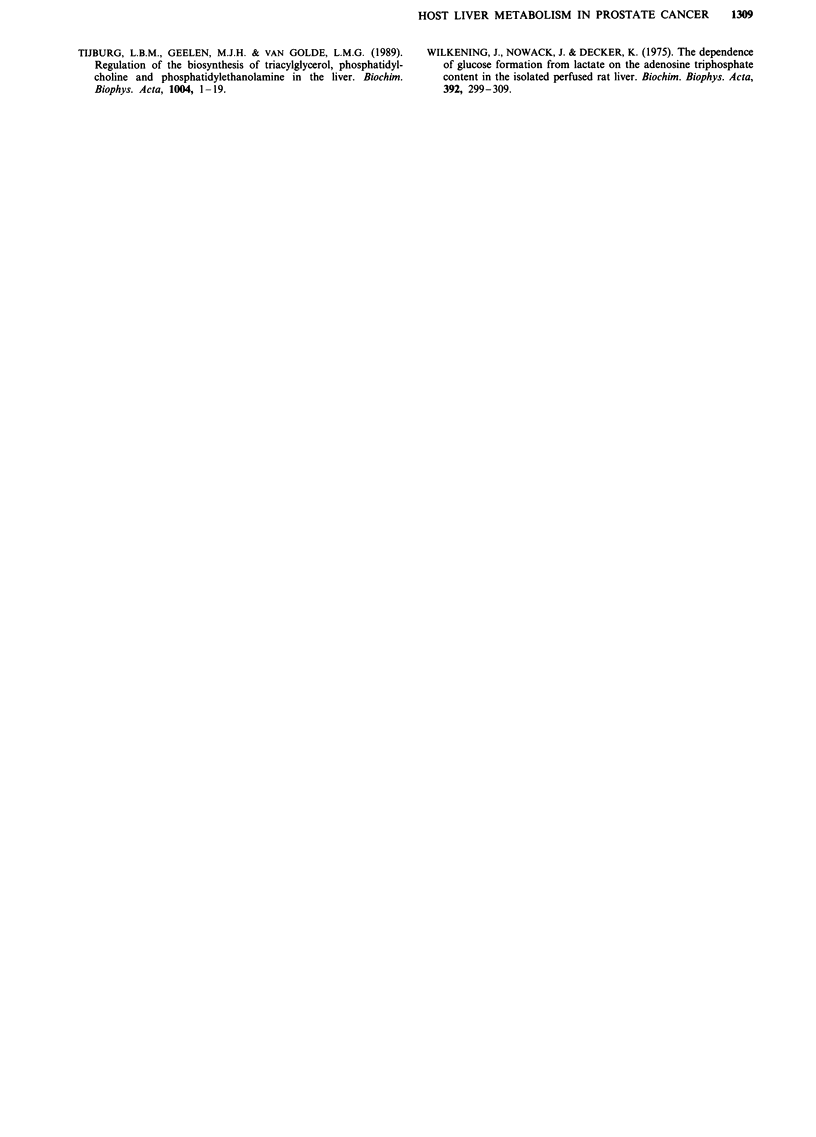# Altered phosphorylation status, phospholipid metabolism and gluconeogenesis in the host liver of rats with prostate cancer: a 31P magnetic resonance spectroscopy study.

**DOI:** 10.1038/bjc.1993.242

**Published:** 1993-06

**Authors:** P. C. Dagnelie, J. D. Bell, S. C. Williams, T. E. Bates, P. D. Abel, C. S. Foster

**Affiliations:** Institute of Internal Medicine II, Erasmus University of Rotterdam, The Netherlands.

## Abstract

**Images:**


Br. J. Cancer (1993), 67, 1303–1309 © Macmillan Press Ltd., 1993

### Altered phosphorylation status, phospholipid metabolism and gluconeogenesis in the host liver of rats with prostate cancer: a $^{31}P$ magnetic resonance spectroscopy study

P.C. Dagnelie,[1,2] J.D. Bell,[2] S.C.R. Williams,[5] T.E. Bates,[6] P.D. Abel[3] & C.S. Foster[4]

[1]*Institute of Internal Medicine II, Erasmus University of Rotterdam, PO Box 1738, 3000 DR Rotterdam, The Netherlands;* [2]*NMR Unit,* [3]*Department of Urology, and* [4]*Department of Histopathology, Royal Postgraduate Medical School, Hammersmith Hospital, London W12 0HS, UK;* [5]*NMR Facility, Department of Chemistry, Queen Mary and Westfield College, London E1 4NS, UK;* [6]*Department of Neurochemistry, Institute of Neurology, London WC1N 3BG, UK.*

**Summary** $^{31}P$ magnetic resonance spectroscopy (MRS) *in vivo* and *in vitro* was used to study modulation of host liver (HL) metabolism in rats bearing the MAT-LyLu variant of the Dunning prostate tumour. Animals were inoculated either with $10^6$ or $10^7$ MAT-LyLu cells, or with saline to serve as controls. Carcass weight in tumour-bearing (TB) animals decreased despite similar food and water intake in both groups. Absence of metastatic tumour cells from HL of all TB animals was confirmed by histological examination. Twenty-one days after inoculation, $^{31}P$ MRS showed a 2.5-fold increase in [Pi]/[ATP] ratios in HL *in vivo* ($P<0.001$) which was confirmed by $^{31}P$ MRS of liver extracts *in vitro* ($P<0.005$). Phosphodiester to ATP ratios were significantly increased ($P<0.05$) in HL *in vivo*, but absolute PDE levels were similar in both groups. Phosphomonoester to ATP ratios did not change, although absolute phosphomonoester levels in HL were reduced by −41% (not significant). In HL extracts *in vitro*, sharp reductions in the levels of glucose-6-phosphate ($P<0.05$), fructose-6-phosphate ($P=0.05$), phosphocholine ($P<0.001$), glycerophosphocholine ($P<0.001$), and glycerophosphoethanolamine ($P<0.001$) were observed. Electron microscopy revealed increased amounts and altered distribution of rough endoplasmic reticulum in HL. These findings show that experimental prostate cancer significantly affects hepatic phosphorylation status, phospholipid metabolism, and gluconeogenesis in the host animal, and demonstrate the value of combined MRS *in vivo* and *in vitro* in monitoring HL metabolism in cancer.

In the USA and the United Kingdom, prostate cancer is the third leading cause of male death from malignant disease. A generalised increase in the incidence of prostatic cancer has occurred throughout the world during the past 25 years so that it is now greater than 30% in men over 50 years of age and rises to 80% by age 80 years. At the time of diagnosis, approximately 75% of patients already have either locally extensive or metastatic disease. Presently, no reliable tumour-markers are available with which to assess, independently, functional differentiation within human prostatic cancers or to determine the effect a particular malignancy will exert on an individual patient (Foster, 1991).

It has been known for many years that the cancer-bearing state is generally associated with profound alterations in host metabolism, contributing significantly to the syndrome of cancer cachexia and ultimately death (Lawson *et al.*, 1982; Kern & Norton, 1988; Rossi-Fanelli *et al.*, 1991). Many of these aberrations, including increased Cori cycle activity, increased protein turnover and hyperlipidemia, relate to changes in the host liver (i.e. liver without metastatic disease). Thus, in the host liver of tumour-bearing (TB) animals, a reduced phosphorylation state (Argilés & López-Soriano, 1991) as well as increases in energy expenditure (Roh *et al.*, 1985), gluconeogenesis (Noguchi *et al.*, 1989; Liu *et al.*, 1990), and protein synthesis (Ternell *et al.*, 1988) were reported. Increased hepatic phospholipid concentrations relative to triglyceride concentrations were also observed (Nakazawa & Mead, 1976). These alterations are associated with marked changes in liver enzyme activities and prominent reexpression of fetal isoenzymes (Herzfeld & Greengard, 1972, 1977).

One approach to investigating the relationship between metastatic phenotype and host status is by means of a reliable tumour-model. The Dunning R-3327 rat prostate cancer model resembles the human disease in its appearance, metastatic behaviour and ability to produce multiple sublines of varied phenotype. The original tumour was derived from a Copenhagen rat prostatic dorsal lobe carcinoma (Dunning, 1963) and proved to be serially transplantable into the flanks of both Copenhagen and Copenhagen/Fisher F1 hybrid rats (Lubaroff *et al.*, 1980). Of the several well characterised behavioural phenotypes that have been cloned from this tumour, the MAT-LyLu subline rapidly metastasizes to lymph nodes and lungs, but not to the liver (Isaacs & Coffey, 1983).

In the present study, we used the MAT-LyLu subline in order to investigate the effect of prostatic cancer on the metabolism of the liver as a major host organ, using $^{31}P$ magnetic resonance spectroscopy (MRS) *in vivo* and *in vitro*. $^{31}P$ MRS is a non-invasive technique which has been previously used in the study of hepatic physiology and disease (Meyerhoff *et al.*, 1990; Oberhaensli *et al.*, 1990; Cox *et al.*, 1992*a*). $^{31}P$ MRS can detect a wide range of metabolites, including intermediates of phospholipid metabolism and glycolysis/gluconeogenesis, adenine nucleotides, and Pi (Radda *et al.*, 1989).

### Materials and methods

### Animal preparation

MAT-LyLu rat prostate carcinoma cells were obtained from Dr J. Isaacs (Johns Hopkins Cancer Center, Baltimore, USA) and cultured as previously described (Bashir *et al.*, 1990). At confluence, cells were harvested using 0.025% trypsin in 10 mM sodium phosphate buffer (pH 7.4) without $Ca^{2+}$ or $Mg^{2+}$. In two subsequent experiments, adult male Copenhagen-Fisher F1 hybrid rats (Harlan-Olac Ltd, Bicester, Oxford, UK) were inoculated into subcutaneous tissues of the left flank with either $1\times10^7$ (experiment 1, $n=3$) or $1\times10^6$ (experiment 2, $n=6$) tumour cells suspended in 500 µl of sterile saline. Apart from the number of inoculated cells, the design of the two experiments was

Correspondence: P.C. Dagnelie, Institute of Internal Medicine II, Erasmus University of Rotterdam, PO Box 1738, 3000 DR Rotterdam, The Netherlands.

identical. Control animals were inoculated with saline (experiment 1: $n = 4$, experiment 2: $n = 5$). Thereafter, animals were maintained in adjacent individual cages with water and standard rat chow (Pilsbury's Ltd, Birmingham, UK) available *ad libitum*. Body weight, and food and water intake were monitored daily. Carcass weight was calculated by subtracting weight of primary and metastatic tumours from total body weight. At 21 days after inoculation, animals were fasted overnight and anaesthetised using $0.5\ ml\ kg^{-1}$ Hypnorm[R] (Janssen Pharmaceutical Ltd, Grove, Oxford, UK; containing 0.315 mg fentanyl citrate and 10 mg fluanisone per ml), and $1\ mg\ kg^{-1}$ Hypnovel (Midezolam; Roche Laboratories, Division of Hoffmann-La Roche Inc, Nutley, N.J. 07110). A midline laparotomy was performed and a two turn, 14 mm diameter coil was placed directly onto the liver. Animals were maintained at 37°C throughout the experiment via a heated pad.

*MRS* in vivo

MRS data were acquired *in vivo* using a SIS-200 MR imaging spectrometer interfaced to an Oxford Instruments 4.7 Tesla, 30 cm bore superconducting magnet. In order to optimise the magnetic field homogeneity in the region of the coil, the coil was transmission line tuned to 200.06 MHz for detection of $^{1}H$ MRS signals. Typical linewidths of 50 Hz were observed for the water resonance. $^{31}P$ MRS data were acquired with the coil tuned to 80.98 MHz, using a pulse of approximately 45° at the center of the coil, and repetition time (TR) of both 1.5 and 8 s (256 and 64 signal averages, respectively). Spectral quantitation of $^{31}P$ MRS data was carried out in a similar manner as described previously by Cox *et al* (1992*a*), after baseline correction for the broad phospholipid signal as described in Bates *et al.* (1989). Data were weighted by line broadening of 20 Hertz prior to Fourier transformation.

*MRS* in vitro

On completion of MRS *in vivo*, part of the liver was freeze-clamped at liquid nitrogen temperature (Bates *et al.*, 1988). In experiment 1, the liver was removed prior to freeze-clamping, whereas in experiment 2, liver tissue was freeze-clamped *in situ* in order to prevent ischaemia. Therefore, for the measurement of concentrations of adenine nucleotides, inorganic phosphate, and sugar phosphates, only data from experiment 2 were included in this paper. Tissue samples were extracted with perchloric acid (Bates *et al.*, 1988), and adjusted to pH = 7.5 using potassium hydroxide. The resulting supernatant was lyophilised and redissolved in $D_2O$. After adding EDTA to a final concentration of ca. 100 mM, pH was readjusted to 7.5. High-resolution $^{31}P$ MRS spectra were acquired on a 8.4 Tesla Bruker system, using a 35° excitation pulse with TR 10 s. Proton scalar coupling interactions were removed by using low power proton decoupling. Phosphocreatine was added as an internal chemical shift reference and concentration standard.

*Microscopy*

For light microscopy, specimens were fixed in fresh neutral-buffered formalin (10% v/v). After routine embedding in paraffin wax, tissue sections were cut at 3 μm and stained with hematoxylin and eosin. For electron microscopy, 1 mm-cubes of tissue were fixed in 2% glutaraldehyde in sodium phosphate-buffer (pH 7.2, 0.2 M). Tissue blocks were post fixed in 1% osmium tetroxide in Millonig's buffer (Millonig, 1961) 1 h at room temperature. Thereafter, following two washes for 30 min each in distilled water, blocks were dehydrated in graded alcohols followed by three changes of TAAB[R] resin (TAAB Laboratories, Aldermaston RG7 4QW, UK) for 1 h each. Tissue blocks were embedded in polyethylene capsules (BEEM capsules, TAAB Laboratories, address as above) in fresh identical resin. Polymerisation was performed at 60°C overnight. Blocks were cut at 1 μm on a Reichert ultramicrotome using a glass knife. Ultrathin sections of selected areas were then cut at 80 nm using a diamond knife. Sections were taken onto uncoated nickel grids and stained in saturated methanolic urinyl acetate for 3 min followed by Reynold's lead citrate (Reynolds, 1963) for 8 min, both at room temperature. After drying, sections were examined and photographed using a Philips CM10 electron microscope at 80 kV.

*Statistical analysis*

Results are expressed as means ± s.e.m. in μmol per *g* wet weight and analysed for significance by Student's *t*-test for independent groups. For data not distributed normally (i.e. Pi/ATP ratios *in vivo* and *in vitro*), Wilcoxon's rank sum test is used. *P*-values $\leqslant 0.05$ are considered significant. For data from experiment 2 only, absolute phosphomonester (PME) and phosphodiester (PDE) concentrations *in vivo* were calculated by multiplying [PME]/[APT] and [PDE]/[ATP] ratios *in vivo* at TR = 8 s by ATP concentrations as measured *in vitro*. The validity of this calculation is supported (1) by the notion that all intracellular ATP in rat liver is MRS-visible *in vivo* (Iles *et al.*, 1985; Desmoulin *et al.*, 1987), and (2) by optimisation of our protocol *in vivo* which indicated that a TR of greater than or equal to 8 s gave the same metabolite ratios.

### Results

*Animals*

In all host animals, tumours grew subcutaneously at sites of inoculation. No ulceration of overlying skin was seen in any of the animals. At 21 days following inoculation, when the experiments were terminated, all primary tumours were between 3 and 5 cm in diameter. Food and water intake of TB animals were within 10% of the control values, and no relation between these variables and test results was observed. Total body weight at the end of the study was similar in both groups of animals, but carcass weight of TB animals had decreased by 13 ± 2% (mean ± s.e.m.) during the 21-day study period, whereas body weight of NTB animals had increased by 7 ± 2% (difference TB *vs* NTB animals: $P < 0.001$).

*Histology*

Histological examination revealed no metastatic tumour cells to be present in multiple sections of the livers from TB animals, despite the growth of metastatic tumours up to 1 cm diameter in abdominal lymph nodes and within lung parenchyma. No difference in cell volume or cell density of hepatocytes was observed between TB and NTB animals.

*MRS* in vivo

$^{31}P$ MRS spectra *in vivo* from the liver of TB animals (host liver) and NTB animals (control) revealed no significant difference in signal-to-noise (Figure 1 a,b). Spectra (Figure 1, Table I) showed a 2.5-fold increase in [Pi]/[ATP] ratios in the host liver as compared to control liver. Phosphodiester (PDE) to ATP ratios in host liver were significantly increased (+ 28%), but absolute PDE levels in host and control liver were similar (mean ± s.e.m.: 4.54 ± 1.00 *vs* 4.48 ± 0.56 μmol per *g* wet weight). No changes in [PME]/[ATP] were observed. Although absolute PME levels in the host liver were reduced by 40%, the difference from control values did not reach statistical significance (4.01 ± 1.00 *vs* 6.85 ± 1.47 μmol per *g* wet weight, $P > 0.05$).

*MRS* in vitro

$^{31}P$ MRS of liver extracts *in vitro* (Figure 2, Table II) showed a 2.1-fold increase in [Pi]/[ATP] ratios in the host liver. ATP concentrations were reduced by 28% in the host liver, but the

**Figure 1** $^{31}$PMRS spectra *in vivo* **a**, the host liver of a tumour-bearing rat and **b**, the liver of a control rat. Peak assignments: α-ATP, β-ATP, γ-ATP: α-, β-, and γ-phosphate groups of ATP; PDE, phosphodiesters; PME, phosphomonoesters.

difference as compared to control liver was not significant ($P > 0.05$). No changes in [ADP] and [AMP] were detected. Marked reductions in phosphocholine (− 49%), glycerophosphocholine (GPC, − 76%) and glycerophosphoethanolamine (GPE, − 85%) concentrations were observed in the host liver (Figure 2, Table III), but phosphoethanolamine concentrations were not altered. Glucose-6-phosphate (G6P) concentrations in the host liver were decreased by 63% and fructose-6-phosphate (F6P) concentrations by 73% (Table IV). No significant changes in concentrations of 3-phosphoglycerate, glyceraldehyde-3-phosphate or sn-glycerol-3-phosphate were observed ($P > 0.05$).

**Table I** Metabolite concentrations *in vivo* in the host liver of tumour-bearing rats ($n = 9$) and the liver of control rats ($n = 9$), as determined by $^{31}$P MRS. Values shown are means ± s.e.m. Abbreviations: PDE, phosphodiesters; PME, phosphomonoesters

| | *Tumour-bearing* | *Control* |
|---|---|---|
| [Pi]/[ATP] | 1.83 ± 0.32 | 0.74 ± 0.06[a] |
| [PME]/[ATP] | 1.47 ± 0.14 | 1.57 ± 0.22 |
| [PDE]/[ATP] | 1.62 ± 0.12 | 1.27 ± 0.09[b] |

Tumour-bearing *vs* control. [a]$P < 0.001$ (Wilcoxon's rank sum test). [b]$P < 0.05$ (*t*-test).

Within the TB group, MRS findings *in vivo* and *in vitro* were not significantly related with tumour load or carcass weight ($P > 0.05$). Phosphocholine and ATP concentrations *in vitro* were significantly correlated ($r = 0.83$, $P = 0.001$). Furthermore, the sum of phosphocholine, G6P and F6P concentrations as detected *in vitro* was significantly correlated with PME levels *in vivo* ($r = 0.79$, $P < 0.001$).

**Figure 2** $^{31}P$ MRS spectra of tissue extract *in vitro* of **a**, host liver of tumour-bearing rats and **b**, liver of control rats. Spectra shown are $^{31}P$ MRS spectra from mixed samples (**a**: $n = 7$, **b**: $n = 5$). Peak assignments: α-ATP, γ-ATP: α- and γ-phosphate groups of ATP; G6P, glucose-6-phosphate; GPC, glycerophosphocholine; GPE, glycerophosphoethanolamine; NTP/NDP, nucleotide triphosphates and diphosphates; PC, phosphocholine; PCr, phosphocreatine (added as an internal chemical shift reference and concentration standard); PE, phosphoethanolamine; X, extraction artefact.

**Table II** Concentrations, determined by $^{31}P$ MRS *in vitro*, of adenine nucleotides and inorganic phosphate in the host liver of tumour-bearing rats ($n = 6$) and the liver of control rats ($n = 5$). Values shown are means ± s.e.m.

| *Absolute concentration* | *Tumour-bearing ($\mu mol\ g^{-1}$ wet weight)* | *Control ($\mu mol\ g^{-1}$ wet weight)* |
|---|---|---|
| ATP | 2.55 ± 0.40 | 3.53 ± 0.29 |
| ADP | 1.37 ± 0.24 | 1.54 ± 0.28 |
| AMP | 0.47 ± 0.12 | 0.32 ± 0.07 |
| Pi | 11.13 ± 2.40 | 8.43 ± 1.90 |
| *Relative concentration* | | |
| Pi/ATP | 4.93 ± 1.20 | 2.32 ± 0.41[a] |

Tumour-bearing *vs* control: [a]$P < 0.05$ (Wilcoxon's rank sum test).

**Table III** Concentrations, determined by $^{31}P$ MRS *in vitro*, of intermediates of phospholipid metabolism in the host liver of tumour-bearing rats ($n = 9$) and the liver of control rats ($n = 9$). Values shown are means ± s.e.m.

| | *Tumour-bearing ($\mu mol\ g^{-1}$ wet weight)* | *Control ($\mu mol\ g^{-1}$ wet weight)* |
|---|---|---|
| Phosphocholine | 1.48 ± 0.20 | 2.91 ± 0.32[a] |
| Phosphoethanolamine | 0.53 ± 0.09 | 0.76 ± 0.22 |
| Glycerophosphocholine | 0.14 ± 0.06 | 0.58 ± 0.09[a] |
| Glycerophosphoethanolamine | 0.10 ± 0.03 | 0.67 ± 0.10[a] |

Tumour-bearing *vs* control: [a]$P < 0.001$ ($t$-test).

**Table IV** Concentrations, determined by $^{31}P$ MRS *in vitro*, of sugar phosphates in the host liver of tumour-bearing rats ($n = 6$) and the liver of control rats ($n = 5$). Values shown are means ± s.e.m.

| | *Tumour-bearing ($\mu mol\ g^{-1}$ wet weight)* | *Control ($\mu mol\ g^{-1}$ wet weight)* |
|---|---|---|
| Glucose-6-phosphate | 0.12 ± 0.04 | 0.32 ± 0.08[a] |
| Fructose-6-phosphate | 0.17 ± 0.04 | 0.64 ± 0.22[a] |
| Glyceraldehyde-3-phosphate | 0.30 ± 0.12 | 0.24 ± 0.13 |
| sn-Glycerol-3-phosphate | 0.16 ± 0.02 | 0.15 ± 0.04 |
| 3-Phosphoglycerate | 0.29 ± 0.06 | 0.48 ± 0.14 |

Tumour-bearing *vs* control: [a]$P \leqslant 0.05$ ($t$-test).

### *Electron microscopy*

Ultrastructural studies of liver tissue revealed a generalised increase in the amount of rough endoplasmic reticulum (ER) in hepatocytes of livers from TB animals (Figure 3). The subcellular distribution of the ER was also altered, when compared with liver from control animals. In the former, the ER was stacked closely around hepatocyte nuclei from which it extended to infiltrate between and to surround mitochondria. Generally, a much closer apposition of ER and mitochondria was seen in hepatocytes of TB animals than in control hepatocytes. In addition, whereas mitochondrial morphology was generally uniform in control hepatocytes, being predominantly circular in cross-section, the morphology of mitochondria in hepatocytes of TB animals was more variable with many ovoid and elongated forms.

### Discussion

This study has demonstrated modulation of hepatic energy and phospholipid metabolism to occur in the host livers of rats bearing the Dunning MAT-LyLu variant of prostatic cancer but in the absence of liver metastases. These observations confirm specific alterations in subcellular membrane composition and metabolism of the host liver to be a direct consequence of the presence of a malignant neoplasm in the host animal. The metabolic abnormalities observed here by $^{31}P$ MRS *in vivo* are markedly distinct from alterations previously reported in liver disease. Most liver diseases studied by $^{31}P$ MRS *in vivo* were characterised by increased [PME]/[ATP] ratios with or without a reduction [PDE]/[ATP] ratios (Angus *et al.*, 1990; Meyerhoff *et al.*, 1990; Oberhaensli *et al.*, 1990; Cox *et al.*, 1992*a*, 1992*b*). In contrast, in this study we observed an increase in [Pi]/[ATP] and [PDE]/[ATP ratios as well as a tendency for reduced absolute PME concentrations in the host liver of TB animals. The rise in [PDE]/[ATP] was explained by a decrease in [ATP]; absolute PDE levels did not change.

The lack of significance in the apparent fall in PME levels *in vivo* may have been due to errors in estimating this peak.

**Figure 3** Ultrastructural appearances of hepatocytes from **a**, control livers and **b**, host livers of tumour-bearing rats. In each electron micrograph the bar indicates 1 µm. In all the control livers, mitochondria appeared uniform, compact and round with small amounts of adjacent endoplasmic reticulum. Hepatocytes from tumour-bearing animals contained increased numbers of frequently irregularly-shaped mitochondria which were intimately surrounded by relatively increased amounts of endoplasmic reticulum.

Since the PME peak *in vivo* is a multicomponent resonance (Ling & Brauer, 1990), it is also possible that reduced intracellular concentrations of some components of the PME peak (such as phosphocholine, G6P and F6P) were compensated by rising levels of other components (such as AMP) contributing to this peak. Our observation that [PME] measured *in vivo* was significantly correlated with the sum of phosphocholine, G6P and F6P concentrations as measured *in vitro* would suggest that decreased concentrations of these compounds contributed to depressed [PME] *in vivo*.

Although the changes in [Pi]/[ATP] and [PME] resemble alterations observed in normal rat liver after prolonged fasting (Desmoulin *et al.*, 1990), our findings were neither caused by anorexia (as shown by similar food intake by TB and NTB animals), nor by the overnight fast preceding MRS (since control animals were also fasted overnight). The resemblance with MRS spectra of fasted liver is therefore remarkable and might suggest a physiological parallel between fasting and the tumour-bearing state. One possible explanation for this similarity would be the increase of hepatic gluconeogenesis which has been observed in the host liver of TB animals (Shearer *et al.*, 1983; Noguchi *et al.*, 1989; Liu *et al.*, 1990). Since gluconeogenesis has demands on ATP supply, this could contribute to ATP depletion in the fasting state as well as in the host liver. Although ATP levels were decreased in TB animals, they were generally above the critical concentration of 2 µmol per g wet weight below which they could have limited the rate of gluconeogenesis (Wilkening *et al.*, 1975).

An increase in glucose-6-phosphatase activity has been reported in host liver of TB animals (Gutman *et al.*, 1969). Although the relation between metabolic rate and steady state concentrations is not straightforward, such an increase in glucose-6-phosphatase activity could provide a possible explanation for the fall in G6P levels observed in our study.

Concentrations of phosphocholine, GPC and GPE were drastically reduced in the host liver, suggesting alterations in phospholipid metabolism related with the tumour-bearing state. These changes in the host liver do not mimic the alterations observed in tumourous tissue, where increases in phosphocholine, and sometimes also phosphoethanolamine, GPC and GPE levels, are observed (Daly & Cohen, 1989; Podo *et al.*, 1987). In rat mammary tumours, phosphocholine levels were positively but GPC and GPE levels negatively related with indices of cell growth (Smith *et al.*, 1991). Phosphocholine, which is synthesised from choline within ATP the choline kinase reaction, is a precursor for phosphatidylcholine synthesis. There is some evidence supporting a role of the choline kinase reaction in the regulation of phosphatidylcholine synthesis (review in Tijburg *et al.*, 1989). The reported $K_m$ of choline kinase for ATP in rat liver is 3.7 mM (Pelech & Vance, 1984), so that the low ATP levels observed in host liver (range 1.27–4.03 mM) could have been rate-limiting for this reaction. This hypothesis is supported by the high correlation observed between phosphocholine and ATP concentrations in host liver.

The cause of the decrease in GPC and GPE concentrations is uncertain. GPC and GPE are produced by hydrolysis of phosphatidylcholine and phosphatidylethanolamine, respectively, but little is known about their metabolic function. Elevated GPC and GPE levels in mouse tumours were reduced after antitumour treatment, and the concomitant rise in glycerol-3-phosphate levels suggested increased GPC and GPE breakdown (Podo *et al.*, 1987). However, in spite of marked reductions in GPC and GPE levels in the host liver, we did not observe an increase in glycerol-3-phosphate concentrations in this organ.

The PDE resonance *in vivo* contains contributions from GPC and GPE as well as from phospholipid headgroups of subcellular membranes (Bates *et al.*, 1989), especially the ER (Murphy *et al.*, 1989). Assuming that the water-soluble compounds GPC and GPE are MRS-visible *in vivo*, in our study they may account for a maximum of 5% of the PDE signal *in vivo* in host liver, and 28% in control liver. Since total PDE levels in host and control liver were not different, this implies that the contribution of the ER to the PDE signal was probably increased in the host liver. The increased size of ER in the host liver detected by ultrastructural studies would provide an explanation for this higher intensity of the

MRS signal derived from the ER. The increased ER is also compatible with the elevated hepatic phospholipid content which was reported in the host liver of human patients with various tumours (Nakazawa & Yamagata, 1971) and later confirmed in a mouse model (Nakazawa & Mead, 1976). The increased proximity of rough ER to hepatocyte mitochondria identified by electron microscopy in TB animals (Figure 3) supports the notion of an enhanced level of physical and functional contact between these two organelles.

In conclusion, we have used a well-characterised rat model of prostate cancer to investigate the effects of this malignancy on the host liver – an organ not directly involved by the neoplasm. The study has shown clear structural and metabolic alterations to occur in a reproducible and predictable manner within the liver. This study not only supports earlier reports of an increased [Pi]/[ATP] ratio in the host liver of rats with transplanted sarcomas (Schneeberger et al., 1989) but it emphasises MRS as a valuable and non-invasive modality with which to monitor and to improve the management of patients with prostatic and other malignancies.

We should like to thank Sue Keefe and Ashley Reynolds for assistance in the design and implementation of the experiments. P.C.D. was supported by fellowships from the Dutch Cancer Society and the Royal Netherlands Academy of Arts and Sciences.

### References

ANGUS, P.W., DIXON, R.M., RAJAGOPALAN, B., RYLEY, N.G. & 4 others (1990). A study of patients with alcoholic liver disease by $^{31}$P nuclear magnetic resonance spectroscopy. *Clin. Sci.*, **78**, 33–38.

ARGILÉS, J.M. & LÓPEZ-SORIANO, F.J. (1991). The energy state of tumor-bearing rats. *J. Biol. Chem.*, **266**, 2978–2982.

BASHIR, I., SIKORA, K. & FOSTER, C.S. (1990). Cell-surface oligosaccharides expressed by phenotypically distinct sublines of the Dunning 3323 rat prostate cancer. *Biochem. Soc. Trans.*, **18**, 968–969.

BATES, T.E., WILLIAMS, S.R., BUSZA, A.L. & GADIAN, D.G. (1988). A $^{31}$P nuclear magnetic resonance study *in vivo* of metabolic abnormalities in rats with acute liver failure. *NMR Biomed.*, **1**, 67–73.

BATES, T.E., WILLIAMS, S.R. & GADIAN, D.G. (1989). Phosphodiesters in the liver: the effect of field strength on the $^{31}$P signal. *Magn. Res. Med.*, **12**, 145–150.

COX, I.J., MENON, D.K., SARGENTONI, J. & 8 others (1992*a*). Phosphorus-31 magnetic resonance spectroscopy of the human liver using chemical shift imaging techniques. *J. Hepatol.*, **14**, 265–275.

COX, I.J., BELL, J.D., PEDEN, C.J., ILES, R.A., FOSTER, C.S., WATAPANA, P. & WILLIAMSON, R.C.H. (1992*b*). *In vivo* and *in vitro* phosphorus-31 magnetic resonance spectroscopy of focal hepatic malignancies. *NMR Biomed.*, **5**, 114–120.

DALY, P.F. & COHEN, J.S. (1989). Magnetic resonance spectroscopy of tumors and potential *in vivo* clinical applications: a review. *Cancer Res.*, **49**, 770–779.

DESMOULIN, F., COZZONE, P.J. & CANIONI, P. (1987). Phosphorus-31 nuclear-magentic-resonance study of phosphorylated metabolites compartmentation, intracellular pH and phosphorylation state during normoxia, hypoxia and ethanol perfusion in the perfused rat liver. *Eur. J. Biochem.*, **162**, 151–159.

DESMOULIN, F., CANIONI, P., MASSON, S., GEROLAMI, A. & COZZONE, P.J. (1990). Effect of ethanol on hepatic energy metabolism and intracellular pH in chronically ethanol-treated rats. A $^{31}$P NMR study of normoxic of hypoxic perfused liver. *NMR Biomed.*, **3**, 132–138.

DUNNING, W.F. (1963). Prostate cancer in the rat. *Natl Cancer Inst. Monogr.*, **12**, 351–369.

FOSTER, C.S. (1991). Predictive factors in prostatic hyperplasia and neoplasia. *Human Pathol.*, **21**, 575–577.

GUTMAN, A., THILO, E. & BIRAN, S. (1969). Enzymes of gluconeogenesis in tumor-bearing rats. *Isr. J. Med. Sci.*, **5**, 998–1001.

HERZFELD, A. & GREENGARD, O. (1972). The dedifferentiated pattern of enzymes in livers of tumor-bearing rats. *Cancer Res.*, **32**, 1826–1832.

HERZFELD, A. & GREENGARD, O. (1977). The effect of lymphoma and other neoplasms on hepatic and plasma enzymes of the host rat. *Cancer Res.*, **37**, 231–238.

ISAACS, J.T. & COFFEY, D.S. (1983). Model Systems for the Study of Prostatic Cancer. *Clin. Oncol.*, **2**, 479–498.

ILES, R.A., STEVENS, A.N., GRIFFITHS, J.R. & MORRIS, P.G. (1985). Phosphorylation status of liver by $^{31}$P-n.m.r. spectroscopy, and its implications for metabolic control. *Biochem. J.*, **229**, 141–151.

KERN, K.A. & NORTON, J.A. (1988). Cancer cachexia. *J. Parent. Ent. Nutr.*, **12**, 286–298.

LAWSON, D.H., RICHMOND, A., NIXON, D.W. & RUDMAN, D. (1982). Metabolic approaches to cancer cachexia. *Ann. Rev. Nutr.*, **2**, 277–301.

LING, M. & BRAUER, M. (1990). *In vitro* $^{31}$-NMR spectroscopic studies of rat liver subjected to chronic ethanol administration. *Biochim. Biophys. Acta.*, **1051**, 151–158.

LIU, K.J.M., HENDERSON, T.O., KLEPS, R.A., REYES, M.C. & NYHUS, L.M. (1990). Gluconeogenesis in the liver of tumor rats. *J. Surg. Res.*, **49**, 179–185.

LUBAROFF, D.M., CANFIELD, L. & REYNOLDS, C.W. (1980). The Dunning Tumours. In *Models of Prostate Cancer*, Murphy, G.P., (ed.) pp.243–263, A.R. Liss: New York.

MEYERHOFF, D.J., KARCZMAR, G.S. & WEINER, M.W. (1990). Abnormalities of the liver evaluated by $^{31}$P MRS. *Invest. Radiol.*, **24**, 980–984.

MILLONIG, G. (1961). Advantages of a phosphate buffer for $OsO_4$ solutions in fixation. *J. Appl. Phys.*, **32**, 1637 (abstract B26).

MURPHY, E.J., RAJAGOPALAN, B., BRINDLE, K.M. & RADDA, G.K. (1989). Phospholipid bilayer contribution to $^{31}$P NMR spectra *in vivo*. *Magn. Res. Med.*, **12**, 282–289.

NAKAZAWA, I. & YAMAGATA, S. (1971). Biochemical changes of the lipid in biopsied livers of patients with malignant neoplastic diseases. *Tohoku J. Exp. Med.*, **103**, 129–139.

NAKAZAWA, I. & MEAD, J.F. (1976). Effect of transplanted human ovarian cancer tissue on liver lipid metabolism of nude mice. *Lipids*, **11**, 159–161.

NOGUCHI, Y., VYDELINGUM, N.A. & BRENNAN, M.F. (1989). The reversal of increased gluconeogenesis in the tumor-bearing rat by tumor removal and food intake. *Surgery*, **106**, 423–431.

OBERHAENSLI, R., RAJAGOPALAN, B., GALLOWAY, G.J., TAYLOR, D.J. & RADDA, G.K. (1990). Study of human liver disease with P-31 magnetic resonance spectroscopy. *Gut*, **31**, 463–467.

PELECH, S.L. & VANCE, D.E. (1984). Regulation of phosphatidylcholine biosynthesis. *Biochim. Biophys. Acta.*, **779**, 217–251.

PODO, F., CARPINELLI, G., DI VITO, M., GIANNINI, M., & 5 others (1987). Nuclear magnetic resonance analysis of tumor necrosis factor-induced alterations of phospholipid metabolites and pH in Friend leukemia cell tumors and fibrosarcomas in mice. *Cancer Res.*, **47**, 6481–6489.

RADDA, G.K., RAJAGOPALAN, B. & TAYLOR, D.J. (1989). Biochemistry *in vivo*: an appraisal of clinical magnetic resonance spectroscopy. *Magn. Reson. Q.*, **5**, 122–151.

REYNOLDS, E.S. (1963). The use of lead citrate at high pH as an electron-opaque stain in electron microscopy. *J. Cell. Biol.*, **17**, 208–212.

ROH, M.S., EKMAN, L.G., JEEVANANDAM, M. & BRENNAN, M.F. (1985). Elevated energy expenditure in hepatocytes from tumor-bearing rats. *J. Surg. Res.*, **38**, 407–415.

ROSSI-FANELLI, F., CASCINO, A. & MUSCARITOLI, M. (1991). Abnormal substrate metabolism and nutritional strategies in cancer management. *J. Parent. Ent. Nutr.*, **15**, 680–683.

SCHNEEBERGER, A.L., THOMPSON, R.T., DRIEDGER, A.A., FINLEY, R.J. & INCULET, R.I. (1989). Effect of cancer on the *in vivo* energy state of rat liver and skeletal muscle. *Cancer Res.*, **49**, 1160–1164.

SHEARER, J., CALDWELL, M., CROSBY, L.O., MILLER, E., BUZBY, G.P. & MULLEN, J.L. (1983). Tumor effects on gluconeogenesis in the isolated perfused rat liver. *J. Parent. Ent. Nutr.*, **7**, 105–109.

SMITH, T.A.D., ECCLES, S., ORMEROD, M.G., TOMBS, A.J., TITLEY, J.C. & LEACH, M.O. (1991). The phosphocholine and glycerophosphocholine content of an oestrogen-sensitive rat mammary tumour correlates strongly with growth rate. *Br. J. Cancer*, **64**, 821–826.

TERNELL, M., ZACHRISSON, H. & LUNDHOLM, K. (1988). RNA polymerase activity and protein synthesis in mouse tumor-host liver compared to benign para-neoplastic reactions. *Int. J. Cancer*, **42**, 464–469.

TIJBURG, L.B.M., GEELEN, M.J.H. & VAN GOLDE, L.M.G. (1989). Regulation of the biosynthesis of triacylglycerol, phosphatidylcholine and phosphatidylethanolamine in the liver. *Biochim. Biophys. Acta*, **1004**, 1–19.

WILKENING, J., NOWACK, J. & DECKER, K. (1975). The dependence of glucose formation from lactate on the adenosine triphosphate content in the isolated perfused rat liver. *Biochim. Biophys. Acta*, **392**, 299–309.